# Postoperative Pressure-Induced Alopecia and Perioperative Care: A Case Report

**DOI:** 10.7759/cureus.88351

**Published:** 2025-07-20

**Authors:** Yamei Yun, Chao Nian, Na Liu

**Affiliations:** 1 Nursing, Plastic Surgery Hospital, Chinese Academy of Medical Sciences and Peking Union Medical College, Beijing, CHN

**Keywords:** care, operation, perioperative, postoperative, pressure alopecia

## Abstract

Postoperative pressure alopecia is a rare complication whose incidence is positively correlated with the duration of surgery. Here we showed a case about pressure alopecia one month after a lengthy operation. The patient suffered for the hair loss over her occiput measuring about 3 × 4 cm. This case reminds us that primary prevention of pressure alopecia is need to be emphasized, as this can minimize unnecessary distress to patients. We should pay attention to this kind of intraoperative complication, because it is entirely possible to prevent it from occurring through our practice.

## Introduction

Postoperative pressure alopecia is a rare complication whose incidence is positively correlated with prolonged surgical procedures. It presents as a local alopecia and is easily confused with other types of alopecia if the medical staff do not take into account the relevant surgical background. It usually occurs in adults but is rarely seen in children [1]. Although most pressure alopecia can be self-healing, usually within two to four weeks, short-term alopecia can be stressful for both the patients and the plastic surgeons. Circumscribed pressure alopecia usually occurs in occiput or head vertex, which are often the most vulnerable areas to compression during surgery, especially after a lengthy surgical procedure with prolonged immobilization of the head (usually more than three hours should be considered vulnerable), such as Whipple surgery, cardiovascular surgery, etc. [2-4]. Plastic surgery usually lasts for a short time; therefore, postoperative pressure alopecia cannot be paid attention to by plastic surgeons and anesthesiologists. However, some plastic surgeries last for a relatively long time, such as breast reconstruction or microsurgical operation, which increases the complications associated with anesthesia. Though hair has no vital physiologic function, but is important to our self-image. Any unpleasant outcomes, such as hair loss, that occur in patients, especially for those who ask for cosmetic or reconstructive surgery, are not desirable. Therefore, it is necessary to summarize similar cases, lest we increase unnecessary distress to patients and plastic surgeons.

## Case presentation

**Figure 1:**
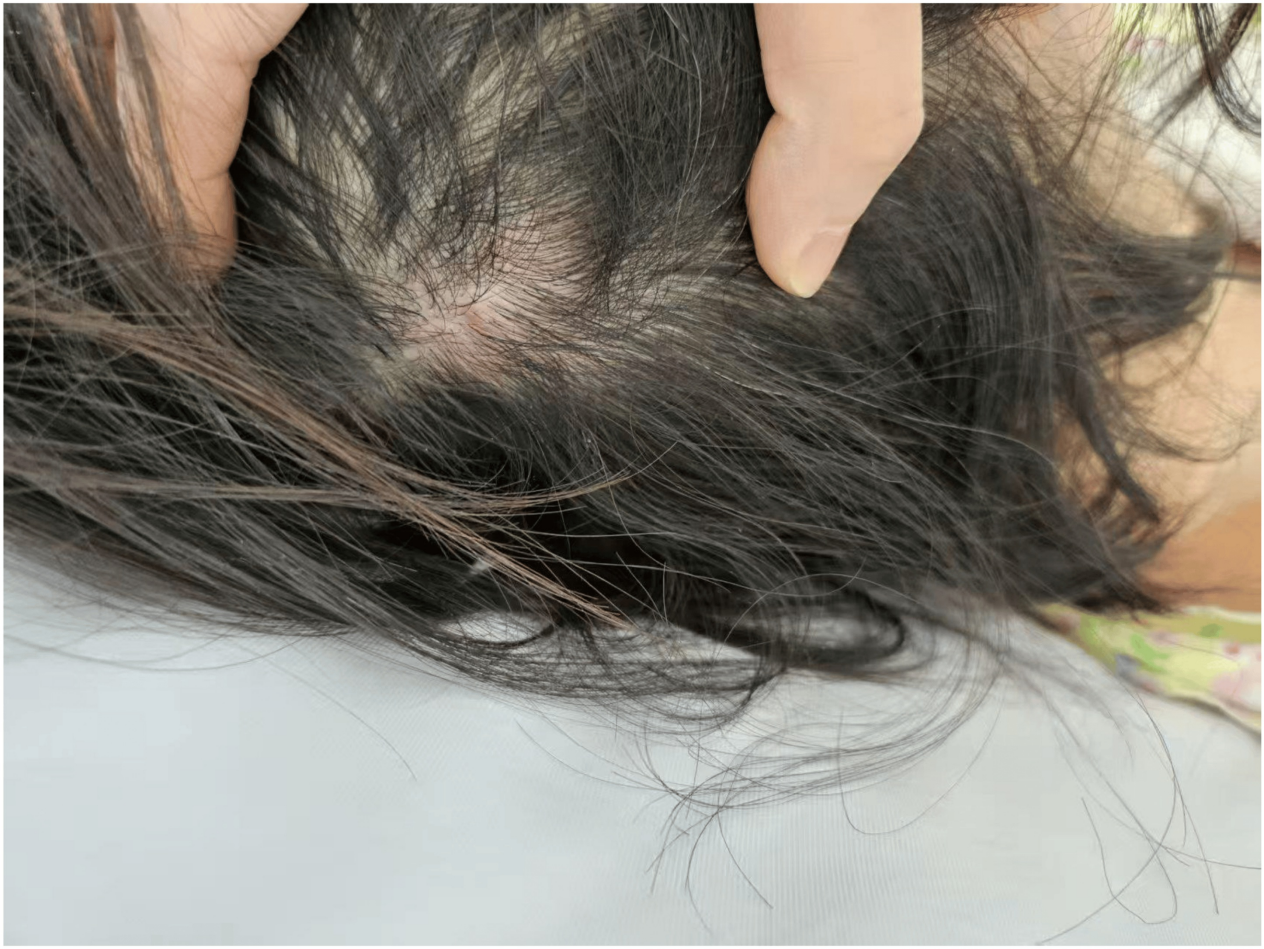
Postoperative photograph showing redness and swelling in the right occipital area. The patient reported pain in the affected region.

A 43-year-old, 50 kg (body mass index [BMI] 19.53) female with a medical history of left breast cancer underwent reconstruction of the left breast using a Deep Inferior Epigastric Perforator (DIEP) flap. She did not have any underlying autoimmune or psychiatric conditions. There was no preceding history of scalp disease or any comorbidities that might be associated with alopecia. First, the patient lay supine on the surgical table without the use of a gel donut headrest. Then, after general anesthesia was administered, the anesthetist placed a cotton roll under the patient’s neck to tilt the head back at approximately a 10-degree angle throughout the operation, to broaden the surgical field of vision and provide appropriate neck support. The duration of anesthesia was 835 minutes, and the operating time was 820 minutes. During this procedure, the patient's head was briefly lifted five times for about two seconds each time. As documented in the anesthesia notes, her systolic blood pressure ranged from 90 to 110 mmHg and diastolic pressure from 50 to 60 mmHg. When she recovered from the anesthetic, she immediately complained of pain on the right occiput and found redness and swelling in the right occipital area (Figure 1). 

**Figure 2:**
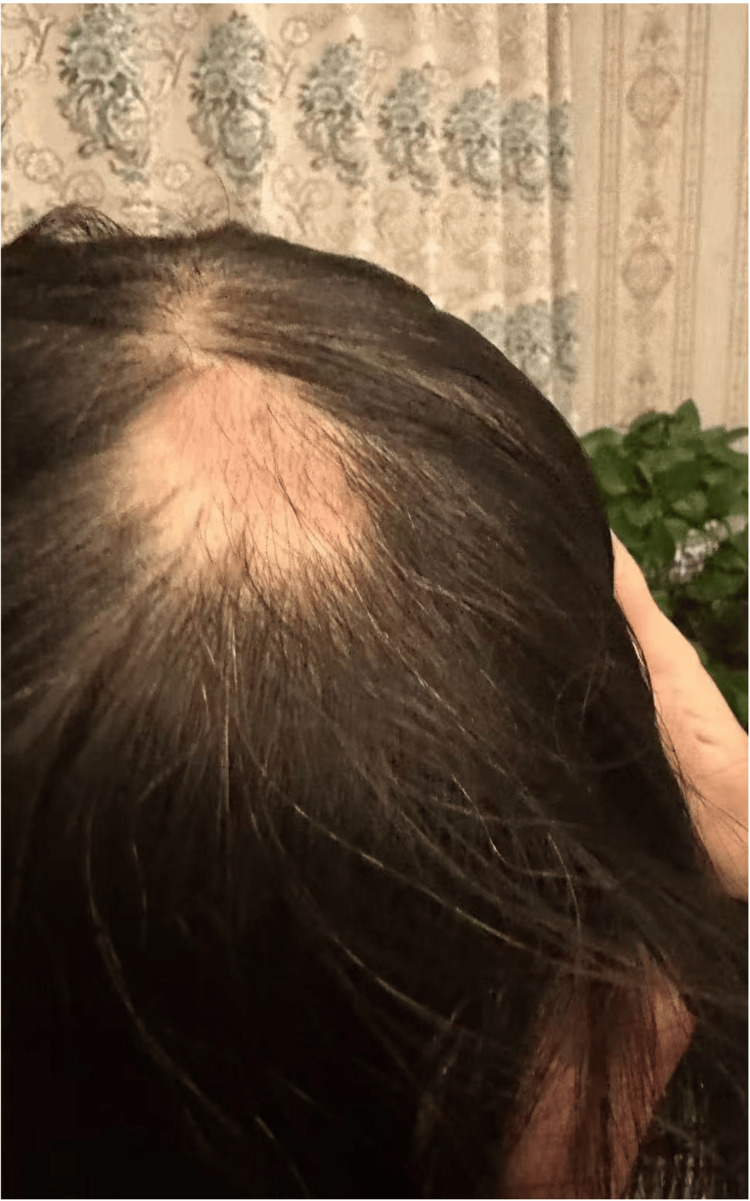
A 43-year-old woman presented with complete hair loss in the occipitoparietal area one month after left breast reconstruction using a DIEP flap. The alopecic area measured 3 × 4 cm. DIEP, Deep Inferior Epigastric Perforator

Her postoperative recovery period went smoothly, and she was discharged on the ninth postoperative day. No other symptoms were recorded postoperatively after one month, when the hair loss was first noticed (Figure 2).

The hair loss over her occiput was complete in an area measuring approximately 3 × 4 cm. Following consultation with a senior dermatologist, a diagnosis of alopecia was made. The patient provided informed consent for publication of this case.

## Discussion

Many cases of postoperative pressure alopecia have been documented in the literature. It may present within the first postoperative week as a swollen, painful area on the scalp, though most cases appear within one month of surgery [5]. The etiology of postoperative pressure alopecia is believed to be positively correlated with the duration of the operation or hospitalization. Patients who have been under anesthesia with head immobilization for more than three hours should be considered at high risk for postoperative pressure alopecia [6]. In our patient, the duration of anesthesia was 835 minutes, which is far above three hours, and she complained of pain on the right occiput and noticed swelling in the right occipital area immediately post-operation, which is the most likely time of pressure alopecia. This is suggestive of having local compromised capillary circulation that may have contributed to the development of alopecia. At this moment, we can be able to increase the blood supply by using infrared rays or teach the patient to massage the scalp to prevent alopecia as perioperative nursing care.

Among the reasons for pressure alopecia, any prolonged pressure concentrated on a small area, such as prolonged head immobilization, can cause scalp ischemia and potentially lead to hair loss [7], especially under anesthesia when the patient lacks self-protective mechanisms to maintain tissue perfusion at the microcirculation level. It is worth noting that our patient had her head repositioned downward during the operation to prevent prolonged pressure on the occipital area, but the duration of each repositioning was short, and the head was moved only about five times throughout the procedure. So, what is the effective way to move the head during operation, and how often to be moved needs to be discussed. Through a search of the literature, intermittent repositioning of the head at half-hour intervals is a good way to reduce the incidence of pressure alopecia, and perioperative rotation of the head is another good way to reduce pressure alopecia [7-9]. In addition, scalp blood perfusion is often considered to be very good, but what is the critical weight and time point that can cause temporary blockage of blood vessels in the weight-bearing scalp? That is, how much force and for how long can lead to scalp ischemia severe enough to make recovery difficult? The above questions need further research. In addition, other risk factors such as controlled hypotension and massive blood loss during the operation can aggravate local tissue ischemia [10]. To our surprise, other risk factors referred to in previous literature include prolonged endotracheal intubation, and certain surgical positions may also aggravate local tissue ischemia, potentially leading to alopecia, even permanent alopecia [11].

Intraoperative measures like tilting the head back are the way that many anesthesiologists use to maintain the airway patent during surgery. However, such a practice increases positional risk, including continued pressure over a specific area of the scalp [12]. There is a hypothesis that this position causes temporary occlusion of blood vessels, leading to hypoxia, vasculitis, and subsequent alopecia. Therefore, it’s important to remind anesthesiologists not to use an excessively high cotton pad to tilt the head back. Or we can use a pillow or something like head headrest to protect the compression site. As for whether to use a headband, the current view is that using hard or semi-hard gel headrests for prolonged periods (over 3 hours), including head rings or gel donut headrests, is not recommended, as they may increase the incidence of postoperative pressure alopecia. Some research suggests that all pressure sites should be well-padded with soft pillows or other appropriate pads during long-duration operations, and that frequent checks should be made for any signs of injury [2].

## Conclusions

Any intraoperative complication, such as postoperative pressure alopecia, can result in patient concern or distress postoperatively, although some of them can eventually return to normal. Awareness is the key to prevention. It reminds us that primary prevention needs to be emphasized, as this can minimize unnecessary distress to patients. We should pay attention to this kind of intraoperative complication, because it is entirely possible to prevent it from occurring through our practice.
